# First person – Matthew Penaso-Stinson and Summer Paulson

**DOI:** 10.1242/bio.062112

**Published:** 2025-07-02

## Abstract

First Person is a series of interviews with the first authors of a selection of papers published in Biology Open, helping researchers promote themselves alongside their papers. Matthew Penaso-Stinson and Summer Paulson are co-first authors on ‘ Macrophages migrate persistently and directionally upon entering 2D confinement in the presence of extracellular matrix’, published in BiO. Matthew and Summer conducted the research described in this article as PhD students in Dr Jeremy Rotty's lab at Uniformed Services University of the Health Sciences, Bethesda. Matthew is now a postdoc in the lab of Dr Martin Meier-Schellersheim at National Institutes of Health (NIH), Bethesda, investigating how binding of distinct extracellular matrix fibers determines macrophage directional migration and inflammatory responses. Summer is a recent PhD graduate interested in articulating the interaction between the microenvironment and intracellular signaling that drives microglial function.


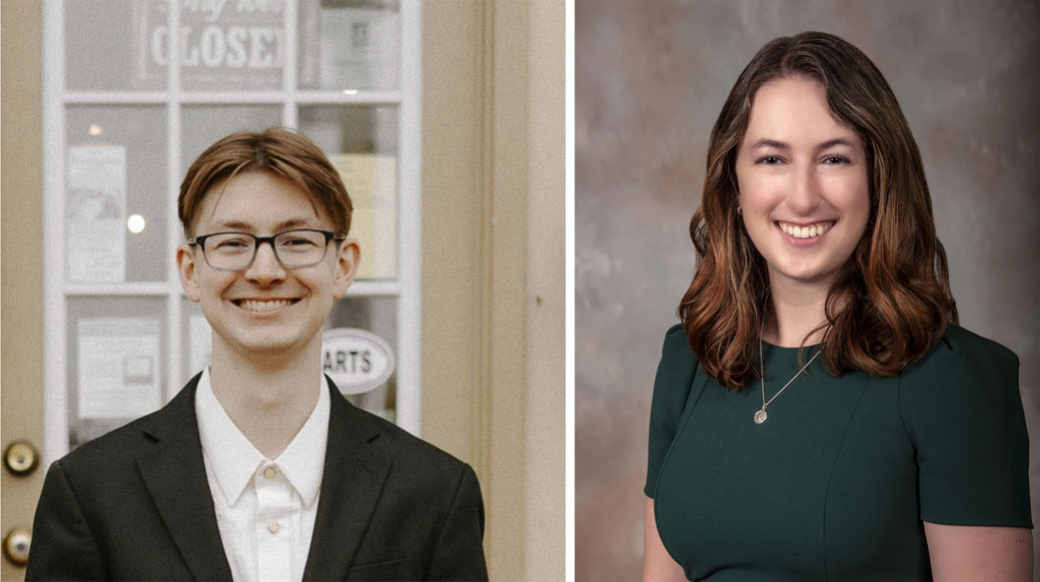


**Matthew Penaso-Stinson; Summer Paulson**.

**Describe your scientific journey and your current research focus**

**M.P.S.:** I began my scientific journey with a bachelor's degree in biology from McDaniel college, where I studied the mRNA decapping protein DCP2 during my summer research experience with Dr Susan Parrish. After college, I obtained my PhD in molecular and cellular biology in Dr Jeremy Rotty's lab, where I focused on macrophage migration, morphology, and adhesion in different microenvironmental contexts. Currently, I am working on an *in vitro* model system to measure macrophage responses to directional and inflammatory stimuli in a basement membrane-like environment.

**S.P.:** I started my scientific journey with a BS in biological sciences, with a double major in biochemistry and neuroscience, at the University of Minnesota. Intrigued with the more biochemical/cell signalling side of brain function, I sought out opportunities to study any component of that through multiple lab volunteer positions during undergrad. I then spent a year in the GREP postbacc fellowship program at Mayo Clinic studying the potential for prolonging Alzheimer's Disease symptom onset timelines in the lab of Dr Mi-Hyeon Jang, before making my way to Maryland and the Uniformed Services University for the Health Sciences for graduate school. There, I recently obtained my PhD in neuroscience in Dr Jeremy Rotty's lab, focusing on microglial interaction with the surrounding microenvironment and finding new ways to articulate these environmental components in *in-vitro* assays. I also have been focusing on the role the actin cytoskeleton plays with facilitating this response to the microenvironment.

**Who or what inspired you to become a scientist?**

**M.P.S.:** I grew up around science. My dad worked for a small biotech company until I was in high school, and most weekend errands with my family would involve a stop at the lab before going shopping. I enjoyed playing with pipettes and using agarose to make cell models for school projects. I intuited that biology could be the career for me when I enjoyed increasingly difficult molecular biology classes taught by my favourite professor, Dr Susan Parrish, and relished my summer research experience with her.

**S.P.:** I was very interested in the brain growing up, but I specifically remember my interest in research stemming from taking AP Biology in high school and loving the gel electrophoresis PCR experiment they let us do one day in class. At that point, I knew I wanted to have a job that let me “play with the scientist tools”, and it was my high school chemistry teacher who pointed me towards an undergrad major in biochemistry and a job in research. From there, throughout undergrad and graduate school, every day at the lab bench was a fun day, and I enjoyed working on new experiments and new techniques.

**How would you explain the main finding of your paper?**

Macrophages are an innate immune cell that are responsible for fighting infections and repairing damaged tissue. They are highly responsive to their environments, being constantly signalled to by changes in tissue stiffness, directional cues, and other factors. The way that macrophages respond to these competing and potentially conflicting factors to migrate efficiently towards their goal is not fully understood. We have shown that two factors, induced confinement and a surface-bound directional cue, separately, and through different mechanisms, enhance directionality. When combined, macrophage directionality is even further enhanced.

**What are the potential implications of this finding for your field of research?**

There are three main takeaways from our research. First, macrophages migrating from an unconfined region in the body to a confined one will tend to maintain the direction they migrate in. Second, that a surface-bound gradient cue will further enhance this directionality. And third, the Arp2/3 complex is responsible for sensing this gradient under confinement (like it does in 2D), but it is dispensable for confinement-induced directionality. Altogether, we present a simplified and reproducible method to generate under-agarose haptotactic gradients and demonstrate that multiple signalling factors influence macrophage directionality.

**Figure:**
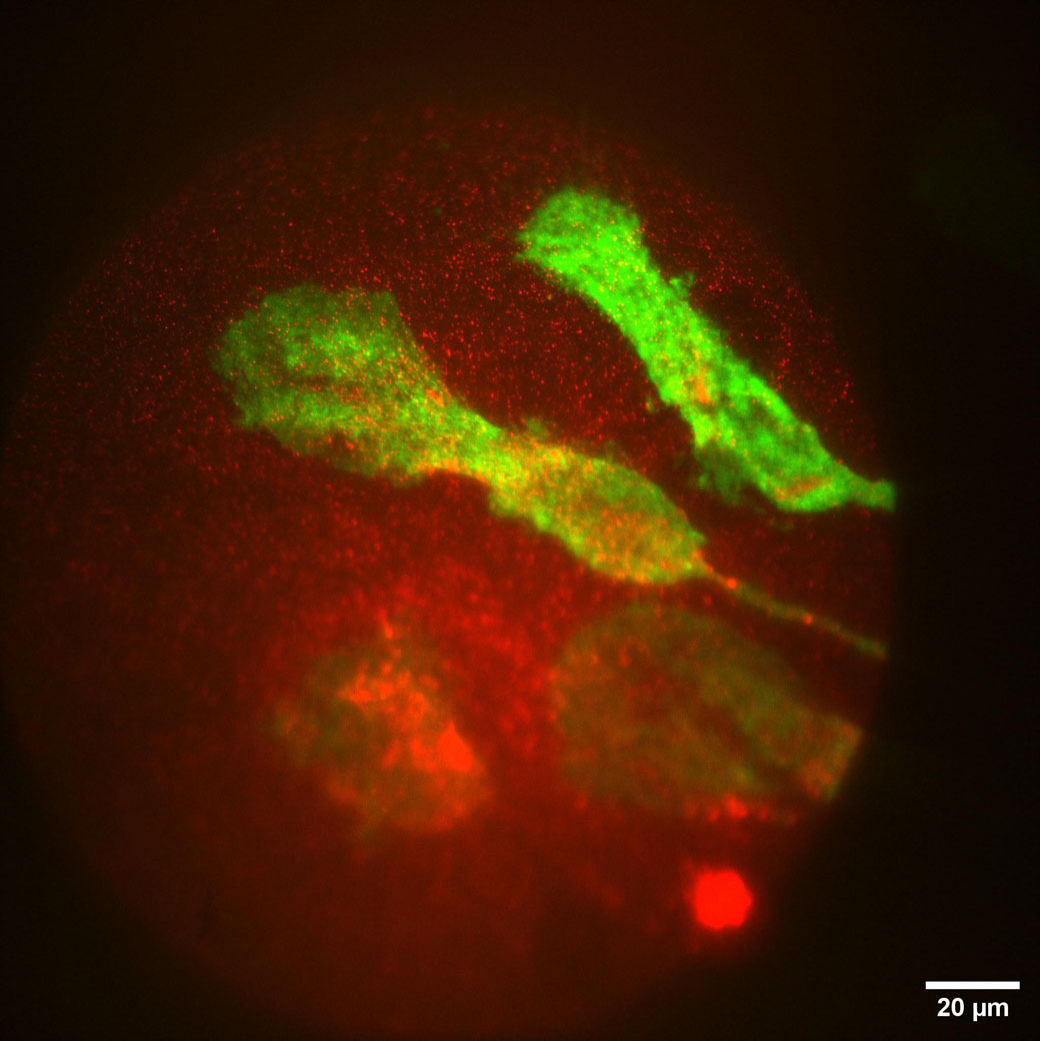
TIRF microscopy showing murine macrophages (green) under agarose-induced confinement binding fibronectin (red) coating the chamber surface.

**Which part of this research project was the most rewarding?**

The most rewarding part for both of us was finally getting our new method to consistently work. There was a lot of trial and error to determine optimal tracking timescales, imaging regions, haptotactic cue concentration, surface coating temperature, and cell-well punch locations. Once it became established, however, the rest of the paper came together nicely.

**What do you enjoy most about being an early-career researcher?**

**M.P.S.:** I enjoy the feeling of getting more and more confident at beginning projects while still working under a mentor guiding my work and thinking. At this stage, I believe it is important to be able to feel comfortable taking calculated risks where there are many experienced lab members who can help and give feedback.

**S.P.:** For me, it would have to be that there has been a lot of groundwork in science already established by the scientists before us, allowing for more creativity at this early stage in what can you bring new to the table. It opens the door to asking more intricate questions and focusing on small details, as we can now work to connect the dots of already existing science.

**What piece of advice would you give to the next generation of researchers?**

“Find your niche” - science has established a lot of large hubs of information, but finding the part that interests you that bisects two or more of those hubs opens you up to helpful, different ways of thinking. A cell biologist does not always approach a problem the same way an immunologist or a neuroscientist might. Finding something that allows you to draw on problem solving skills from multiple scientific communities allows you to take advantage of existing science and drive your research forward more to fill the gaps in our understanding.

**What's next for you?**

**M.P.S.:** I am hoping to continue my research on macrophage responses to complex stimuli *in vitro* and further understand the decision making that impacts their motility and immune signalling. I am able to pursue this research as a postdoc at NIH, and I would love to continue generating more methods and leveraging microscopy to better understand many of these transient behaviours.

**S.P.:** I just recently defended my thesis, and am now in the process of interviewing for a postdoc position while I briefly remain in my thesis lab to finish up the remaining revisionary experiments from my outstanding thesis publications. I would love to continue working on better ways to measure the interaction of the microenvironment and the intracellular signalling in cells to facilitate specific functional outputs, looking with microglia to better study the brain and understand what role these cells play in homeostasis.
